# Pravastatin Administration Alleviates Kanamycin-Induced Cochlear Injury and Hearing Loss

**DOI:** 10.3390/ijms23094524

**Published:** 2022-04-20

**Authors:** Chang Ho Lee, Jiwon Jeon, So Min Lee, So Young Kim

**Affiliations:** Department of Otorhinolaryngology-Head & Neck Surgery, CHA Bundang Medical Center, CHA University, Seongnam 13496, Korea; hearwell@gmail.com (C.H.L.); tw7682@nate.com (J.J.); lws6812@naver.com (S.M.L.)

**Keywords:** hearing loss, pravastatin, aminoglycosides, ototoxicity

## Abstract

The effect of statins on aminoglycoside-induced ototoxicity is controversial. This study aimed to explore the role of pravastatin (PV) in kanamycin-induced hearing loss in rats. Adult rats were intraperitoneally treated with 20 mg/kg/day of kanamycin (KM) for 10 days. In the PV- and PV + KM-treated rats, 25 mg/kg/day of PV was intraperitoneally administered for 5 days. The auditory brainstem response (ABR) thresholds were measured before and after drug treatment using a smartEP system at 4, 8, 16, and 32 kHz. Cochlear changes in poly ADP-ribose (PAR) polymerase (PARP), PAR, and caspase 3 were estimated using Western blotting. PV administration did not increase the ABR thresholds. The KM-treated rats showed elevated ABR thresholds at 4, 8, 16, and 32 kHz. The PV + KM-treated rats demonstrated lower ABR thresholds than the KM-treated rats at 4, 8, and 16 kHz. The cochlear outer hair cells and spiral ganglion cells were relatively preserved in the PV + KM-treated rats when compared with that in the KM-treated rats. The cochlear expression levels of PARP, PAR, and caspase 3 were higher in the KM-treated rats. The PV + KM-treated rats showed lower levels of PARP, PAR, and caspase 3 than the KM-treated rats. PV protected cochleae from KM-induced hearing loss in rats. The regulation of autophagy and apoptosis mediated the otoprotective effects of PV.

## 1. Introduction

Aminoglycosides induce irreversible hearing loss by activating oxidative stress, and the resulting inflammation induces cell death in cochlear outer hair cells and spiral ganglion cells [1]. Gentamicin-induced activation of caspases leads to apoptosis in cochlear hair cells, which was reversed by caspase inhibitors in a study using guinea pigs [2]. In addition to activated apoptosis, excessive autophagy leads to cochlear injury resulting in aminoglycoside-induced hearing loss [3]. Controlled or adaptive autophagy promotes cell survival by degrading cellular organelles via autophagosomes [4]. The dysregulated autophagy accelerates apoptosis. Aminoglycosides induce time-dependent accumulation of autophagosomes in the organ of Corti, thereby aggravating auditory thresholds in rats [5]. The inhibition of autophagy using rapamycin improves gentamicin-induced hearing loss [5]. Rapamycin decreases autophagic signaling via the mammalian target of rapamycin (mTOR), a serine-threonine kinase [6].

Statins are inhibitors of 2-hydroxy-3-methylglutaryl-coenzyme A (HMG-CoA) reductase, a rate-limiting enzyme for cholesterol synthesis, and are used to lower cholesterol levels in clinical settings. In addition to their lipid-lowering capacities, statins have pleiotropic functions that relieve inflammation and neurodegeneration [7,8]. Furthermore, statins help preserve the cochlea against noise, aging, and cisplatin treatment [9,10]. In a preclinical study, the otoprotective effect of pravastatin (PV) was observed in noise-induced hearing loss in mice [9]. In addition, a clinical trial demonstrated a lower rate of cisplatin-induced hearing loss with concurrent prescription of atorvastatin in patients with head and neck cancer (adjusted odds ratio = 0.47, 95% confidence intervals = 0.30–0.78) [10]. 

The effects of statins on aminoglycoside-induced hearing loss have been investigated [11,12,13]. An in vitro study demonstrated that simvastatin protected the hair cells of the organ of Corti against gentamicin-induced ototoxicity [13]. On the contrary, simvastatin induced hair loss with a dose–response association in a study using a zebrafish lateral line [12]. The zebrafish lateral line lacks supporting structures, including the stria vascularis, and resembles vestibular hair cells more than cochlear hair cells. Another in vitro and ex vivo study reported decreased cytoplasmic projections in mouse cochlear neuroblasts and primary rat cochlear explants after simvastatin treatment [11]. The neonatal cochleae are vulnerable to drug toxicity, and supplementation with mevalonate can reverse the toxic effects of simvastatin [11]. Thus, the toxic effect of statins were not determined in an in vivo model. To unravel the effects of statins on aminoglycoside-induced ototoxicity, an in vivo study in mammals is warranted. 

We hypothesized that PV could attenuate cochlear injury induced by kanamycin (KM) by alleviating oxidative stress and regulating autophagy. To examine this postulation, rats with KM-induced ototoxicity were compared with those concurrently administered with PV. A hydrophilic statin of PV was chosen because it was less likely to diffuse through nonspecific tissues than lipophilic statins and presented a pleiotropic mechanism different from that of lipophilic statins [14,15].

## 2. Results

**Figure 1:**
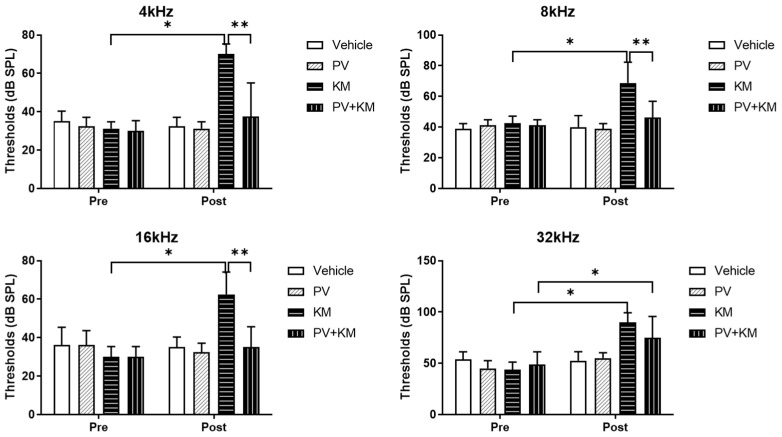
The auditory brainstem response (ABR) thresholds at 4, 8, 16, and 32 kHz of each group were measured using smartEP system. The KM-treated rats demonstrated increased hearing thresholds at 4, 8, 16, and 32 kHz after KM administration, compared with those before drug administration. The PV + KM-treated rats showed lower hearing thresholds at 4, 8, and 16 kHz than the KM-treated rats. (* *p <* 0.05, paired *t*-test between pre- and post-drug treatment; ** *p <* 0.005, unpaired *t*-test between groups).

The hearing levels changed at 4, 8, 16, and 32 kHz after KM treatment (Figure 1). The average auditory brainstem response (ABR) threshold at 4 kHz was 70.0 dB sound pressure level (SPL) (SD = 5.35) in the post-KM-treated rats vs. 31.23 dB SPL (SD = 3.54) in the pre-KM-treated rats (*p <* 0.001). At 8 kHz, the average ABR threshold was 68.75 dB SPL (SD = 4.79) in the post-KM-treated rats vs. 42.50 dB SPL (SD = 1.64) in the pre-KM-treated rats (*p <* 0.001). At 16 kHz, the pre- and post-KM-treated rats had ABR thresholds of 62.50 dB SPL (SD = 11.65) and 30.00 dB SPL (SD = 5.35), respectively (*p* < 0.001). At 32 kHz, the pre- and post-KM-treated rats had ABR thresholds of 90.00 dB SPL (SD = 9.26) and 43.75 dB SPL (SD = 7.44), respectively (*p <* 0.001). The ABR thresholds of the control and PV groups did not differ between pre- and post-treatment at any of the measured frequencies.

**Figure 2:**
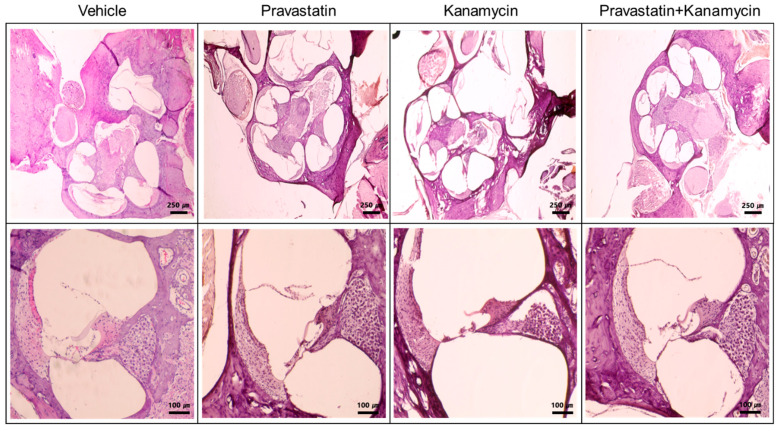
H&E staining of cochleae. The cochleae of the KM group demonstrated loss of outer hair cells and spiral ganglion cells, whereas the cochleae of the PV + KM group showed preserved spiral ganglion cells.

**Figure 3:**
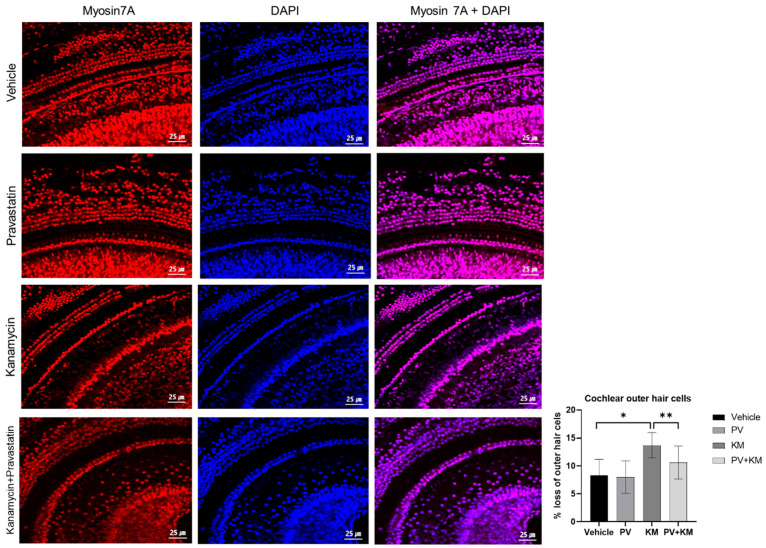
Cochlear whole-mount staining of each group. The KM group showed loss of outer hair cells vs. the control group. The PV + KM group demonstrated less loss of outer hair cells than the KM group (red: myosin 7a-positive cells, blue: DAPI-positive cells, purple: myosin 7a and DAPI-positive cells; * *p <* 0.05, Mann–Whitney U test between the control and KM groups, ** *p <* 0.005, Mann–Whitney U test between the KM and PV + KM groups).

The PV group did not exhibit any changes in hearing levels. Histological examination of cochleae showed that cochlear outer hair cells and spiral ganglion cells were preserved in the PV rats (Figure 2 and Figure 3).

The PV + KM group demonstrated a higher ABR threshold at 32 kHz after drug treatment (75.00 dB SPL [SD = 20.70], *p* = 0.010) than before treatment (48.75 dB SPL (SD = 12.46)). When compared with the KM group, the PV + KM group had lower ABR thresholds at 4, 8, and 16 kHz (37.50 dB SPL (SD = 17.53), *p <* 0.001; 46.25 dB SPL (SD = 10.60), *p* = 0.002; and 35.00 dB SPL (SD = 10.69), *p <* 0.001, respectively). The ABR threshold at 32 kHz did not differ between the KM and PV + KM groups.

The cochleae showed disorientation and loss of outer hair cells and spiral ganglion cells following KM administration (Figure 2). However, the PV + KM-treated rats showed oriented and intact organs of Corti when compared with the KM-treated rats. The KM-treated rats demonstrated a higher loss of outer hair cells when compared with the vehicle rats (13.69% (SD = 2.26) vs. 8.33% (SD = 2.84), *p* = 0.003) (Figure 3). The PV + KM-treated rats showed preserved outer hair cells when compared with the KM-treated rats (13.89% (SD = 2.13) vs. 10.61% (SD = 2.95), *p* = 0.040).

**Figure 4:**
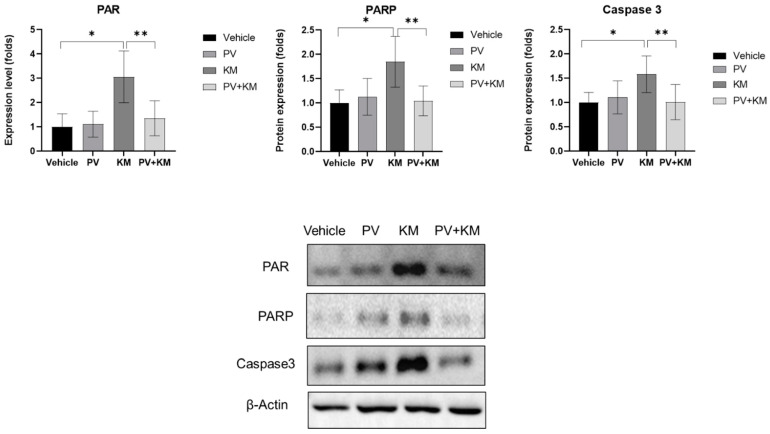
The Western blotting results of PARP, PAR, and caspase 3. The PV + KM group demonstrated lower levels of PARP, PAR, and caspase 3 than the KM group (* *p <* 0.05, Mann–Whitney U test between the control and KM groups; ** *p <* 0.005, Mann-Whitney U test between the KM and PV + KM groups).

The expression of PARP, PAR, and caspase 3 was higher in the KM-treated rats than in the vehicle rats (Figure 4). When compared with the vehicle rats, the KM-treated rats demonstrated 1.85-fold higher levels of PARP (SD = 0.52, *p* = 0.005). The PAR expression was 3.06-fold higher in the KM-treated rats than in the vehicle rats (SD = 1.06, *p* = 0.002). Caspase 3 showed a 1.58-fold higher expression level in the KM-treated rats than in the vehicle rats (SD = 0.38, *p* = 0.008).

The expression levels of PARP, PAR, and caspase 3 were lower in the PV + KM than that in the KM-treated rats: 1.85 (SD = 0.52) vs. 1.04 (SD = 0.31), *p* = 0.009 for PARP; 3.06 (SD = 1.06) vs. 1.00 (SD = 0.54), *p* = 0.002 for PAR; 1.58 (SD = 0.38) vs. 1.00 (SD = 0.21), *p* = 0.008 for caspase 3.

## 3. Discussion

Pravastatin alleviated cochlear injury and hearing loss induced by kanamycin in rat models. Cochlear outer hair cells and spiral ganglion cells were less injured in the PV + KM-treated rats than that in the KM-treated rats. Furthermore, the hearing thresholds were lower at 4, 8, and 16 kHz in the PV + KM-treated rats than those in the KM-treated rats. PV inhibited apoptosis and dysregulated autophagy in the KM-treated rats. The results of the present study improved previous knowledge by identifying the otoprotective effects of PV in an in vivo model of aminoglycoside-induced hearing loss.

In the present study, PARP, PAR, and caspase 3 levels increased following KM administration. PARP is a nuclear enzyme that can be activated by external stimuli such as oxidative stress [16]. PARP activation depletes NAD+ and ATP and accumulates PAR polymer, which in turn binds to AIF and translocates to the nucleus [17]. The energy depletion of NAD+ and ATP can result in necrosis. In addition, PARP-induced PAR accumulation is a characteristic of parthanatos (PARP-dependent cell death). Under oxidative stress, PARP-1 is activated and promotes PAR synthesis in cochlear marginal cells of the stria vascularis [18]. Moreover, recent data have pointed to the mediating role of PARP in autophagy [15]. As PARP, PAR, and caspase 3 were upregulated in the KM-treated rats, it is presumed that multiple regulated cell death mechanisms of apoptosis, parthanatos, and autophagy were activated in KM-induced ototoxicity. In line with the present results, previous research has demonstrated activation of PARP-1 and autophagy after aminoglycoside administration. Streptomycin treatment induces AIF translocation from the mitochondria to the cytoplasm, which activates PARP-1 in neonatal rat cochlear explants [19]. AIF has dual functions of redox regulation in the mitochondria and apoptosis in the nucleus, referred to as necroptosis (programmed necrosis) [20].

PV treatment ameliorated the increased expression of PARP, PAR, and caspase 3 in the KM-induced ototoxicity model in the present study. Similarly, several previous studies have reported the relieving effects of statins on PARP activation and regulation of autophagy [15,21]. PV modulated autophagy through pathways involving PARP in a cell line study [15]. Furthermore, PV protects against dexamethasone-induced avascular necrosis of the femoral head by regulating autophagy [21]. Simvastatin, a lipophilic statin, diminished gentamicin-induced ototoxicity via activation of Akt signaling, which is related to autophagy, in an in vitro study [13]. In addition, PV can protect the cochlea from ototoxic injury by relieving oxidative stress [9]. Statins are thought to relieve oxidative stress by inhibiting the nicotinamide adenine dinucleotide phosphate (NADPH) oxidase complex [22]. By suppressing isoprenylation, statins inhibit the activation of small GTP-binding proteins, which serve as activators of the NADPH oxidase complex [22]. In addition to the protective effects on outer hair cells of cochlea, PV administration improved the spiral ganglion cell population in the present study. To support our findings, an in vitro drug screening study demonstrated another statin, cerivastatin, promoted and regenerated neurites in the mouse spiral ganglia [23].

As statins, including pravastatin, are clinically available drugs, the applications of statins in patients with aminoglycoside-induced hearing loss could be cost-effective. The dose adjustment for the clinical use of pravastatin requires further study. We used a hydrophilic statin, pravastatin, whereas prior studies on the effects of statins mostly used lipophilic statins, such as atorvastatin, simvastatin, and lovastatin [10,11,13,24]. We administered a hydrophilic statin because lipophilic statins have potential ototoxic effects in vitro and ex vivo [11,12]. In addition, lipophilic statins induce a higher risk of adverse effects, such as statin-associated muscle symptoms and neurologic disorders due to non-selective diffusion and solubility across the blood–brain barrier [14,24]. The present study was limited due to the lack of the information on the optimal types and dose of statins on the ototoxicity. Future study will be warranted to delineate the current limitations.

## 4. Materials and Methods

### 4.1. Animal Groups and Noise Exposure

**Figure 5:**
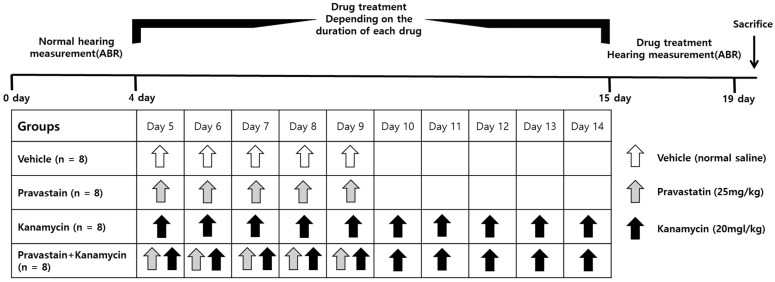
Flow chart of the experiments. Adult rats (8–11 week Sprague–Dawley rats, 200–250 g) were classified into the vehicle, pravastatin (PV), kanamycin (KM), and PV + KM groups (*n* = 8 per group). Rats in the KM group were administered KM (20 mg/kg/day) i.p. for 10 days. Rats in the PV group were administered PV (25 mg/kg/day) i.p. for 5 days. The rats in the PV + KM group were injected with 25 mg/kg/day of PV and 20 mg/kg/day of KM i.p. for the first 5 days, and then with 20 mg/kg/day of KM for 5 days.

This study was approved by the Institutional Animal Care and Use Committee of the CHA University Medical School (IACUC200166). The study followed the guidelines of the Institutional Animal Care and Use Committee of CHA University Medical School. During the study period, standard lab chow and water were provided regularly. The 32 Sprague–Dawley rats (postnatal 8–11 weeks, *n* = 8 per group) were divided into the control, PV, KM, and PV + KM groups (Figure 5). Rats in the PV group were administered PV at 25 mg/kg/day intraperitoneally (i.p.) for the first 5 days. The dose of PV was selected based on the previous study [9]. Rats in the KM group were administered KM at 20 mg/kg/day i.p. for 10 days [25]. The PV + KM-treated rats were injected with 25 mg/kg/day of PV and 20 mg/kg/day of KM i.p. for the first 5 days, followed by 20 mg/kg/day of KM for 5 days. The vehicle rats were treated with equal amounts (50 mL/kg) of normal saline for 10 days. After hearing levels were examined, all rats were euthanized with CO_2_ gas. The cochleae were dissected, and hematoxylin and eosin (H&E) stain (ab245880, abcam, Cambridge, UK) was used for histological examination (cochlear whole mounts, *n* = 2 per group) and Western blotting (*n* = 6 per group).

### 4.2. Hearing Function Tests

The hearing levels were examined before and after drug administration in all rats. The auditory brainstem response (ABR) thresholds at 4, 8, 16, and 32 kHz were estimated (SmartEP, Intelligent Hearing System; Miami, FL, USA) [26] (Figure 2). The reference, ground, and ground electrodes were placed at the vertex, contralateral thigh, and ipsilateral retroauricular area, respectively. Pure tone auditory stimuli of 4, 8, 16, and 32 kHz (duration: 1562 µs, envelope: Blackman, stimulation rate: 21.1/s, amplitude: 90–20 dB SPL, 1024 sweeps) were delivered using an EC1 electrostatic speaker coupled with an earphone. The hearing threshold was set as the lowest sound amplitude that evoked Wave III.

### 4.3. Histological Examination of Cochleae

Cochlear whole mounts (two rats per group) were prepared to examine the morphology of the outer hair cells of the cochlea [27,28]. The cochleae were dissected, and the otic capsule bone was decalcified. Free-floating, dissected cochlear outer hair cells were subjected to immunofluorescent staining. The primary antibodies 1:1000 anti-myosin 7A (Sc74516; Santa Cruz) were incubated overnight at 4 °C. The secondary antibodies 1:2000 Alexa 594 anti-mouse IgG (ab150108; Abcam) and 4′,6-diamidino-2-phenylindole dihydrochloride (DAPI) were incubated for 2–3 h. The cochlear tissues were mounted on slides and imaged using a confocal microscope (Zeiss LSM 880; Zeiss, Land Baden-Württemberg, Germany).

H&E staining (*n* = 2 rats per group) was performed to examine the organ of Corti and spiral ganglion cells [29,30]. Dissected cochleae were embedded in paraffin blocks, and the cochlear blocks were sectioned at a thickness of 10 µm. The slides were stained with H&E solutions (hematoxylin for 5 min and eosin for 45 s). The stained slides were examined using the EVOS^TM^ XL Core Imaging System (#AMEX1000; Invitrogen, Carlsbad, CA, USA).

### 4.4. Western Blotting

The protein expression levels of poly ADP-ribose (PAR) polymerase (PARP), PAR, and caspase 3 were examined in each group of rats (*n* = 4 rats per group). Proteins were extracted from cochlear tissue (Pre-prep, Intron). The protein concentrations were calculated using a microplate reader and compared with bicinchoninic acid (BCA) standards. The quantified equivalent quantities of proteins were resolved using 10% sodium dodecyl sulfate-polyacrylamide gel electrophoresis (SDS-PAGE) in running buffer for 90 min at 80–100 V. Gels were then transferred to polyvinylidene difluoride membranes (Merck Millipore, Burlington, MA, USA) after activation in 20% methanol. SDS-PAGE was conducted in blocking buffer (5% non-fat dry milk in Tris-buffered saline containing Tween-20) for 90 min at 300 mA. The membranes were socked in 1:1000 of anti-PARP (9532S; Cell Signaling Technology, Danvers, MA, USA), anti-PAR (ALX-804-220-R100; Enzo, Farmingdale, NY, USA), and anti-caspase 3 (9662S; Cell Signaling Technology, Danvers, MA, USA) overnight in a cold room (4 °C). After washing with TBST (Tris-buffered saline with 0.1% Tween^®^ 20 Detergent) solution three times, the membranes were incubated with 1:2000 of horseradish peroxidase (HRP)-conjugated secondary antibodies (anti-rabbit IgG, HRP-linked; #7074S, Cell Signaling Technology and goat anti-mouse IgG H&L (HRP); #ab97023, Abcam, Cambridge, UK) for 2 h. After washing with TBST solution, the membranes were activated in an enhanced chemiluminescence kit (Bio-Rad, Hercules, CA, USA) for 1–2 min. The membranes were analyzed using the ImageJ software (National Institutes of Health, Bethesda, MD, USA). Each protein band was estimated, and the intensity of each band was compared with that of β-actin. The protein expression levels in each group were evaluated based on that in the vehicle group.

### 4.5. Statistical Analysis

Hearing levels were analyzed using paired t-tests for each group (pre- vs. post-treatment). After testing for a normal distribution using the Shapiro–Wilk test, cochlear outer hair cell loss and protein expression in each group were analyzed using the Mann–Whitney U test. All data are presented as the average and standard deviation (SD). Statistical significance was set at *p* ≤ 0.05. All analyses were conducted using the SPSS software (ver. 21.0; IBM Corp.; Armonk, NY, USA).

## 5. Conclusions

PV attenuates KM-induced cochlear injury and hearing loss in rats. Moreover, PV may control the dysregulation of apoptosis and autophagy via molecular cascades involving PARP and PAR.

## Data Availability

The data presented in this study are available upon request from the corresponding author.
